# Assessment of the Active Sludge Microorganisms Population During Wastewater Treatment in a Micro-Pilot Plant

**DOI:** 10.3390/bioengineering11121306

**Published:** 2024-12-23

**Authors:** Daniela Roxana Popovici, Catalina Gabriela Gheorghe, Cristina Maria Dușescu-Vasile

**Affiliations:** 1 Chemistry Department, Faculty of Petroleum Refining and Petrochemistry, Petroleum-Gas University of Ploiesti, 100680 Ploiesti, Romania; 2 Petroleum Refining Engineering and Environmental Protection Department, Faculty of Petroleum Refining and Petrochemistry, Petroleum-Gas University of Ploiesti, 100680 Ploiesti, Romania

**Keywords:** microorganisms, bioremediation, pollutants, biodegradation

## Abstract

Knowledge of the impact of chemicals on the environment is important for assessing the risks that chemicals can generate in ecosystems. With the help of pilot-scale micro-tests, it was possible to evaluate the biological sludge in terms of its chemical and biological composition, information that can be applied on an industrial scale in treatment plants. The important parameters analyzed in the evaluation of the biodegradability of wastewater were pH, chemical composition (NH_4_^+^, NO_3_^−^, NO_2_^−^, and PO_4_^3−^), dry substance (DS), inorganic substance (IS), and organic substance (OS), and the biological oxygen demand (BOD)/chemical oxygen consumption (COD) ratio. The examination revealed the presence of free active ciliates *Aspidisca polystyla*, *Lyndonotus setigerum*, *Vorticella microstoma*, fixed by *Zooglee*, *Paramecium* sp., *Opercularia*, *Colpoda colpidium*, *Euplotes*, *Didinum nasutum*, *Stentor*, and *Acineta tuberosa,* metazoa *Rotifers*, filamentous algae, *Nostoc* and *Anabena*, and bacteria *Bacillus subtilis*, *Nocardia*, and *Microccocus luteus.* The novelty of this study lies in the fact that we carried out a study to evaluate the population of microorganisms starting from the premise that the probability of biodegradation of substances is directly proportional to the number of microorganisms existing in the environment and their enzymatic equipment.

## 1. Introduction

Organic substances are artificial pollutants that come from the processing of various substances in refineries (gasoline, diesel, oils, and organic and organic solvents), the chemical industry, or the food industry (albumin, gelatin, keratin, casein, and gluten that, in their molecules, contain nitrogen, sulfur, and phosphorus, in addition to carbon). Under the action of bacteria, organic pollutants are decomposed into simpler products—glycerin and fatty acids, soaps, and waxes. These pollutants come from the textile and paper industry, such as lanolin-type pollutants. Carbohydrates can be found in the simplest products—monosaccharides, disaccharides, and polysaccharides (like dextrin, glycogen, cellulose, or starch)—forming films on the water surface that are challenging to remove. For example, pollution from the oil industry can lower the quality of aquifers, affecting the smell and appearance of the water even at low concentrations. Many aquatic organisms are affected, disrupting the ecological balance. Petroleum products, being lighter than water, form a film on the water’s surface, which hinders oxygenation. As a subsequent effect, the aerobic processes are stopped, with severe consequences. Oil pollutants harm aquatic biocenosis through their toxicity and by blocking air diffusion into polluted water. Inorganic salts increase water salinity, and some can increase water hardness. Increased chlorine levels make the water unsuitable for drinking and industrial use [1,2,3,4,5,6].

The degradability of different micropollutants depends on specific compounds that can remove some impurities. While most impurities can be decomposed in regular purification processes, some substances cannot be eliminated using chemical methods. The ability of organic compounds to biodegrade is affected by the presence of toxic or inhibitory substances that hinder the growth of microorganisms in water [7,8,9,10,11,12].

Biological monitoring of wastewater involves analyzing the composition and diversity of microorganism species in the water. This process is crucial for assessing the impact of wastewater on the environment and for optimizing wastewater treatment processes. Water quality is determined by a conventional set of physical, chemical, biological, and bacteriological characteristics, which are expressed as values and used to categorize the water sample.

Organic pollutants oxidize and decompose in the presence of dissolved oxygen in water. This degradation process is aerobic and involves the consumption of oxygen, leading to the production of carbon dioxide and water (respiration). When oxygen is limited, anaerobic processes, such as denitrification, deamination, and fermentation, occur, leading to the production of unwanted compounds, like hydrogen sulfide and methane. Through denitrification, bacteria can obtain the necessary oxygen for decomposing organic substances. The oxygen dissolved in water is consumed by the microbial decomposition of organic matter as part of cellular metabolic processes.

The process of biological purification involves the use of certain microorganisms that act on toxic or potentially toxic radicals through specific processes: biochemical, metabolic, and bioconversion processes. The effective action of microorganisms in an environment with toxic substances depends on various factors, such as the presence of oxygen, pH levels, contact and retention times, temperature, and the availability of nutrients in the optimal dose. The degradation of toxic compounds works in conjunction with the evolution of certain chemical parameters, such as dissolved oxygen, ammonium, nitrites, and nitrates. This process is aided by the synergistic action of microorganisms present in the biological sludge, including bacteria, protozoa, algae, and rotifers. *Bacillus subtilis* plays an important role in water purification, particularly in biological water and sludge treatment processes. It has the capability to break down complex organic matter, such as proteins, fats, and carbohydrates, into simpler compounds. *Bacillus subtilis* produces various hydrolytic enzymes, like proteases, lipases, and amylases, which aid in the decomposition of organic compounds. Additionally, the enzymes produced by *Bacillus subtilis* and *Nocardia bacteria* accelerate the degradation processes of organic matter, facilitating water purification. These microorganisms can also degrade or transform various pollutants, including hydrocarbons, pesticides, and other toxic substances, into less harmful compounds. *Acinetobacter bacteria* have the enzymatic ability to hydrolyze polyphosphates, transforming them into phosphates. They can break down amino acids, ethanol, and other substances. This process, known as bioremediation, is crucial in reducing pollution in aquatic environments [13,14,15,16,17,18,19].

The research carried out allows a deeper understanding of microbial communities and their ecological functions, thus contributing to the improvement of purification processes and reducing the negative impact of residual substances on the environment. Analysis of the composition and diversity of species in wastewater is essential for the efficient management of water resources and environmental protection. The tests performed focus on the identification of the microbiota that is involved in the purification processes through the degradation of pollutants, the chemical and metabolic transformation of toxics, the elimination of harmful agents, and in efficient water resource management as well.

Knowledge of the purification processes of toxic substances, difficult to remove from the aquatic environment, could be controlled by supplementing the aquatic flora with specific microorganisms that have a high tolerance to the toxic agent. Microorganisms that populate the biological sludge biocenosis serve as sensitive indicators of environmental changes. They can survive and develop in freshwater and marine ecosystems and can be used for environmental risk assessment. These microorganisms can transform substances containing nitrogen and phosphorus from contaminated waters into biomass and bioproducts.

In biological wastewater treatment, understanding the tolerance limits of the microorganisms is necessary. This study focuses on the chemical and biological composition of the biological sludge from the petrochemical industry under laboratory conditions in the micro-pilot plant to evaluate the optimal conditions for the viability of microorganisms. The evaluation of the fauna in the biocenosis of the biological sludge involves microscopic examinations, Gram-stained smears, and identifications on specific culture media. This analysis was conducted following tests carried out in the laboratory between May and July 2024. The chemical and biological composition of the biological sludge from the petrochemical industry is analyzed, and optimal conditions for the efficient elimination of pollutants introduced into the system through synthetic water are established in the micro-pilot plant. The concentrations of pollutants, organic and inorganic substances (IS), as well as pH, ammonium, nitrates, nitrites, and phosphates are calculated. Further analyses are conducted to evaluate the evolution of the sludge volume under laboratory conditions and in the presence of pollutants introduced into the system. The populations of protozoan, metazoan, and specific bacteria microorganisms in the biological sludge biocenosis are also assessed using microscopic examinations, Gram-stained smears, and identifications on specific culture media [20,21,22,23,24,25,26,27,28,29].

The use of chemical substances for the removal of pollutants from wastewater is an energy-consuming process with high costs, with a risk of secondary pollution resulting from chemical interactions between the substances used. Unlike chemical treatment, biological treatment, using microorganisms, is much more efficient, with low energy consumption. The use of microorganisms present in specialized biological sludge for the biodegradation of pollutants from the oil industry with a high content of organic substances (petroleum products) increases the efficiency of the pollutant removal process because they can continuously adapt to oil pollution conditions, without additional costs. Biological treatment can be successfully applied not only in the treatment of wastewater but also for the decontamination of polluted soil, the biological treatment method being sustainable and eco-friendly compared to other methods [30,31,32,33].

The studies conducted aimed to evaluate biological sludge from the point of view of its chemical and biological composition, through pilot-scale treatment of wastewater encountered in an oil refinery. The information has industrial-scale applicability in treatment plants related to the oil industrial area, by supplementing the microflora with specific microorganisms for decontamination [34,35,36].

## 2. Materials and Methods

### 2.1. Presentation of the Micro-Pilot Plant

The research was conducted in the laboratory using a micro-pilot purification system in two stages. The first stage, called the stabilization stage, involved adapting biological sludge collected from a wastewater treatment plant in the petrochemical industry over a period of 14 days. In the second stage, which lasted 21 days, a constant volume of synthetic water (10 mL/L) was introduced into the micro-pilot installation loaded with biological sludge that had been adapted during the stabilization stage [37,38].

**Figure 1:**
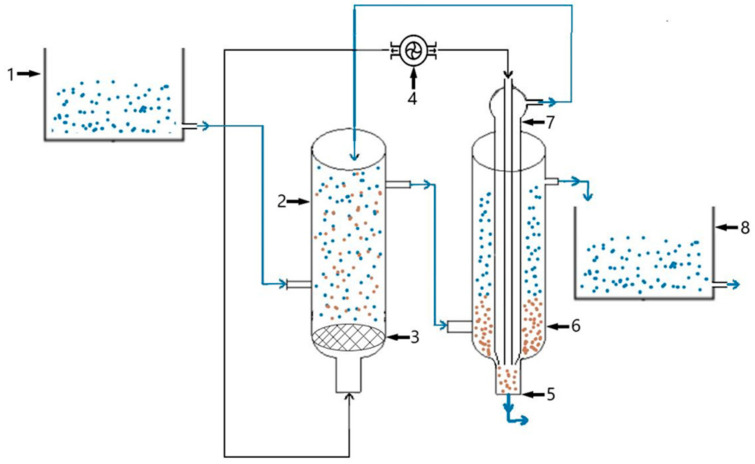
The micro-pilot plant used in the experiments. 1—Food container, 2—aero tank, 3—porous frit, 4—air pump, 5—excess biological sludge discharge spout, 6—decanter, 7—recirculation “gas-lift” system, and 8—collector vessel.

The micro-pilot purification system, depicted in Figure 1, is made of glass and consists of several parts. The supply vessel (1) has a 5 L capacity and is mounted on a support at a height to allow for free-fall supply to the system. In the stabilization stage, the supply vessel was used for nutrient dosing. In the second stage, synthetically polluted water was fed continuously into the supply vessel for 21 days. For the adaptation of the biological sludge in the stabilization stage and to test its evolution in contact with the nutrient solution from the stabilization stage or with synthetic water in the testing stage, a bioreactor (aero tank) (2) with a useful capacity of 700 mL (diameter 7 cm and height 50 cm) was used. At the lower part of the bioreactor, a spigot was provided, through which air was introduced into the system. Above the lower spigot of the bioreactor, a frit with fine pores (3) was mounted, through which oxygen diffused into the biofilter, distributed with the help of a compressor (4). The air distribution was necessary to maintain the viability of the activated sludge with the optimal supplementation of the dissolved oxygen concentration in the system and to homogenize the synthetic water/nutrients/biological sludge contact [11].

The bioreactor is equipped with volume graduations to measure the amount of sludge deposited when aeration is stopped for sludge volume reporting. Below/near the frit, a constant volume of the nutrient solution and synthetic water (10 mL/h) were introduced in the stabilization stage, in the second testing phase, through a supply nozzle. Through the discharge nozzle, water with nutrients (in the stabilization stage) and synthetic water (testing stage) mixed with activated sludge entered the decanter, where settling took place. The settled sludge was recirculated in the air tank with the help of a “gas lift” recirculation pump (7). The decanter (6) has a capacity of 700 mL (diameter 7 cm and height 50 cm) and a nozzle (5) for the evacuation of the sedimented biological sludge. The supernatant released from the pilot system was collected through a discharge spout positioned at the top of the decanter and collected in a collection vessel (8). The connections between the vessels were made with silicone rubber tubing, and the strangulations were made with screw clamps. Assembling the installation and stiffening the system was performed by fixing it on a metal support (Figure 1). The recirculation flow was adjusted to 1 mL/minute.

The water circuit loaded with nutrients/pollutants from the stabilization/testing stages is shown in blue, and the biological sludge circuit is shown in red in Figure 1 [11]. The plant worked continuously during the entire period of the experiments at room temperature (25–30 °C). The feed rate of the reactor was 10 mL/h, and continuous feed mixing in the bioreactor was ensured by a dosing pump through continuous diffusion of air in a concentration of 3 mg/L oxygen. The hydraulic retention time in each bioreactor reaction basin, respectively, in the decanter, was 4.5 days. Taking into account this retention period, we can say that the used water supplied on day 1 was found as purified water on day 9.

### 2.2. Preparation of the Chemical Composition of the Effluent

In the stabilization stage, the nutrient source, denoted as SGM, consisted of glucose (0.1%), peptone (0.1%), diacid potassium orthophosphate (0.1%), complex N:P:K (0.2%), FeSO_4_ (0.05%), and NH_4_Cl (1.5%). The pH was adjusted to 6.5. In the test stage, the impurity source, marked as TSM, was synthetic wastewater with the following chemical composition: NH_4_Cl (5 g/L), MgCl_2_•6H_2_O (0.5 g/L), MgSO_4_•7H_2_O (0.25 g/L), CaCl_2_•2H_2_O (0.25 g/L), NaH_2_PO_4_•2H_2_O (0.3 g/L), NaHCO_3_ (0.3 g/L), KH_2_PO_4_ (1 g/L), KNO_3_ (0.4 g), FeCl_3_• 6H_2_O (0.025 g/L), and FeSO_4_•7H_2_O (0.025 g). The pH was adjusted to 8.5.

The composition of the synthetic water at the beginning of the test had the following chemical composition: 1620 mg NH4^+^/L, 580 mg NO_3_^−^/L, 900 mg PO4^3−^/L, BOD (biological oxygen demand) 250 mg/L, and chemical oxygen demand (COD) 180 mg/L [39,40,41,42,43,44].

The experiment was performed in accordance with the OECD Guideline for Testing of Chemicals-301 [45]. The chemical indicators were accepted at such a high level because the wastewater introduced into the system was constantly dosed in small volumes so that we could observe the dynamics of the population of microorganisms and not destroy the existing microflora. The analysis of the chemical indicators was carried out in accordance with the analysis standards in force.

### 2.3. Preparation of Biological Material and Growth of Biological Cells

During the stabilization and testing phases, the following materials and reagents were used.

Biological sludge obtained from a petroleum refinery in Ploiesti was used as the model organism for experiments at the Petroleum-Gas University of Ploiesti over a 14-day period. The biological sludge was acclimated to laboratory conditions during the stabilization phase using SGM growth medium. For the testing phase, a synthetic water solution, called TSM, was used for 21 days. The biological sludge samples were taken in accordance with SR EN ISO 5667-15:2010 [46] The sample was collected during aeration. The analysis was performed after 30 min rest, after the biological sludge sedimentation [37,38]. An aliquot volume was filtered using the Macherey–Nagel Filter No. MN 640 m. The supernatant was analyzed and NH_4_^+^, NO_3_^−^, NO_2_^−^, PO_4_^3−^—pH, COD, BOD, and DO were determined, and from the filtered part the chemical composition and the weight of the biological suspension, DS, OS, and IS were determined.

The chemical analysis of the composition of the biological sludge collected from the treatment plant before being subjected to the stabilization stage indicated the following parameters: pH 8.2, COD 86 mg/L, BOD 34 mg/L, DS (dry substance) 266 mg/L, DO (dissolved oxygen) 5.2 mg/L, OS (organic substance) 88%, IS (inorganic substance) 12%, 14 mg NH_4_^+^/L, 27 mg NO_3_^−^/L, 8 mg NO_2_^−^/L, and 6.5 mg PO_4_^3−^/L [39,40,41,42,43,44].

Chemical reagents were weighed using an OHAUS model AX224M analytical balance (Ohaus Corporation, Parsippany, NJ, USA). For microscopic examinations, the samples were homogenized using a UNIVERSAL 320R Type 1406-01 centrifuge (Hettich, Beverly, MA, USA). A CELESTRON Microscope (Celestron, Torrance, CA, USA), model 4434, was used to evaluate the biomass. Cell viability was determined through microscopic visualization and quantification in the visual field using a THOMA cell-counting chamber [47,48,49].

A laminar flow hood, incubator, thermostatic oven, homogenizing plate, sterile Pasteur pipettes, sterile test tubes, automatic pipettes with PVC tips, Petri dishes, ultrapure water, sterile cedar oil, physiological serum, and a Gram stain battery (gentian violet, Lugol’s solution, alcohol, acetone, diluted fuchsin (1/10)) were used for the microscopic examination of biological sludge collected from the bioreactor. Nutrient agar culture media were prepared to identify bacterial species in the analyzed biological sludge. The medium’s composition was as follows: bacto-peptone 10 g, sodium chloride 5 g, agar 20 g, meat extract 10 g, and water up to 1000 mL. The mixture was heated to boiling, pH was adjusted to 7.4, and it was filtered and sterilized at 121 °C for 15 min [48].

To facilitate the growth of the bacterium *Microccocus luteus*, specific culture media consisting of three types were prepared: Media A, Media B, and Media C. Media A: meat extract 4 g, proteose-peptone 9 g, sodium chloride 75 g, D-mannitol 10 g, agar 20 g, phenol red 25 mg, and water up to 1000 mL. The second medium, marked “B”, had the following chemical composition: defibrillated blood 100 mL, bacto-peptone 5 g, sodium chloride 5 g, agar 20 g, meat extract 10 g, and water up to 1000 mL. The third medium, marked “C”, had the following composition: meat extract 4 g, proteose-peptone 10 g, sodium chloride 150 g, lactose 15 g, agar 1 g, and water up to 1000 mL. Additionally, lysostaphin (200 µg/mL) was added. After dissolving the reagents, the media were then sterilized at 121 °C for 15 min [48].

To promote the growth of *Bacillus subtilis* bacteria, specific culture media were prepared containing the following reagents: pancreatic casein hydrolysate 10 g, peptic hydrolysate of animal tissue 10 g, lactose 10 g, sucrose 10 g, glucose monohydrate 1 g, iron sulfate (II) 0.2 g, sodium thiosulfate 0.2 g, sodium chloride 5 g, agar 13 g, phenol red 25 mg, and water up to 1000 mL. The components were dissolved in water, the pH was adjusted to 7.4, and they were sterilized at 121 °C for 15 min.

The *Nocardia* bacterium was highlighted using a specific culture medium, as follows: bacto-peptone 10 g, sodium chloride 5 g, agar 20 g, meat extract 10 g, and water up to 1000 mL. The pH was adjusted to 7.4 and it was sterilized at 121 °C for 15 min [48,49,50].

For the laboratory tests, a multi-parameter WTW Inolab MULTI 9630 IDS (WTW, Weilheim, Germany) with three galvanically isolated measuring channels was used, with pH and oxygen measurement. Samples were filtered using 0.45-micron Whatman filter papers. For determining the parameters NH_4_^+^, NO_3_^−^, NO_2_^−^, and PO_4_^3−^, a UV-Vis spectrophotometer, model T85+, PG Instruments, was used.

## 3. Results

During the initial 14-day stage of the experiment, known as the stabilization phase, we worked on adapting the biological sludge sample from the petrochemical industry treatment plant to laboratory conditions. Throughout this phase, we provided the feeding vessel with water containing nutrients to create the best possible living conditions for the biological sludge [51,52].

### 3.1. Plotting Calibration Curves

In the analysis of the supernatant, comprehensive calibration curves were executed for NH_4_^+^, NO_3_^−^, NO_2_^−^, and PO_4_^3−^ indicators. Ultrapure deionized water standards were meticulously prepared from reference solutions. The measurements were rigorously conducted, and the resulting data were thoroughly processed using the renowned Origin 6.0 TM Software Microcal INC 7.0. After graphing the results, the linearity of the response was documented within specific concentration ranges: 0.02–0.2 mg/L for NH_4_^+^ (regression coefficient R^2^ = 0.9998), for NO_3_^−^ (regression coefficient R^2^ = 0.9965) within the concentration range of 0.02–0.2 mg/L, for NO_2_^−^ (regression coefficient R^2^ = 0.9988) within the concentration range of 0.02–0.2 mg/L, and for PO_4_^3−^ within the range of 0.1–1.8 mg/L (regression coefficient R^2^ = 0.9993) [39,40,41,42,43].

### 3.2. Analysis of Biological Material: Determination of Dry Substance (DS), Inorganic Substance (IS), Organic Substance (OS), Biological Oxygen Demand (BOD), and Chemical Oxygen Demand (COD)

Each day, an aliquot volume of 5 mL of sample was taken from the bioreactor after 30 min of settling and after measuring the volume of biological sludge. The collected samples were filtered using filter paper that had been dried in an oven at 105 °C for 2 h and then in a desiccator for 30 min. The filter paper was then dried in an oven at 105 °C for 2 h, placed in a desiccator, and weighed to determine the dry substance (DS) using Equation (1). After weighing, the filter paper was heated to 650 °C for one hour and then weighed again in the desiccator to calculate the ash using Equation (2) [44]:

DS (g/L) = (a × 1000)/V(1)

where:

a = the difference between the weight of the filter paper with the dry substance and the empty filter paper,

V = the sample volume used.

Ash g/L = m_3_ − m_1_/V × 1000 (2)

where:

m_3_ = the weight of the crucible after calcination,

m_1_ = the weight of the empty crucible,

V = the sample volume taken into account.

Finally, the percentages of inorganic and organic matter were calculated using Equations (3) and (4). Equation (3) calculates the percentage of inorganic matter, while Equation (4) calculates the percentage of organic matter:

(IS) = ((RU × 100)/DS), %(3)

where:

DS = dry substance, g/L,

RU = ash g/L.

100 − IS = OS, %(4)

where:

IS = inorganic substance, %,

OS = organic substance, %.

The biological oxygen demand (BOD) measurement of dissolved oxygen (DO) is used by microorganisms for the biochemical oxidation of organic matter due to cellular metabolic reactions. The removal of organic substances in the purification process is quantified by the expression of BOD. Biological oxygen demand (BOD) was analyzed by collecting the aliquot volumes collected from the feed vessel, bioreactor, decanter, and collector vessel. The samples were collected for the initial analysis and a parallel sample that was incubated at 20 °C was analyzed over 5 days. The determination of the content of dissolved oxygen (DO) was calculated using Equation (5) and was carried out in the presence of 2 mL of manganese chloride and 2 mL of iodide-azide mixture, to which 5 mL of sulfuric acid 1:3 was added. The solution was titrated with sodium thiosulfate 0.025 N in the presence of starch:

Dissolved oxygen (DO) = v × f × 0.2/v1 − 4 × 1000, mg/L(5)

where, v = mL of thiosulphate solution used, f = thiosulphate factor, 0.2 = the equivalent in mg of oxygen of a 0.025 N thiosulphate solution, v1 = volume of water analyzed, and 4 = the amount of reagents introduced to fix oxygen.

The biological oxygen demand (BOD) from the analyzed samples was quantified using Equation (6):

BOD = DO A − DO B, mg/L(6)

where DO A = the amount of oxygen present in the sample at the time of collection and DO B is the amount of oxygen present in the sample after 5 days.

The determination of chemical oxygen demand (COD) represents the mass concentration of oxygen equivalent to the amount of potassium dichromate consumed by dissolved and suspended matter. The oxidizable substances in the water were oxidized by potassium bichromate in hot sulfuric acid medium, and the excess bichromate was titrated with Mohr’s salt in the presence of ferroin as an indicator. This analysis quantified the amount of oxygen consumed by the total chemical oxidation of organic compounds to inorganic products. The chemical analysis took place in parallel samples: the analysis of the sample collected from the harvests from the feed vessel, bioreactor, decanter, and the collecting vessel compared to the reference samples of distilled water. The collected sample (1 mL), and in parallel the control sample (distilled water) mixed with mercury sulfate (II), were boiled under reflux for 1 h, with a known amount of potassium dichromate, in the presence of a silver catalyst in a strongly acidified environment with sulfuric acid, so that part of the potassium dichromate was reduced by the oxidizable matter present. The COD value was calculated starting from the amount of reduced potassium dichromate. Taking into account the fact that 1 mole of potassium dichromate is equivalent to 1.5 moles of oxygen (O_2_), the chemical consumption of oxygen was calculated with the following Equation (7):

(7) $$\mathrm{COD}=\frac{8000\cdot c\cdot (V_{1}-V_{2})}{V_{0}},\mathrm{mg}/L$$

where c is the concentration of the amount of substance of the solution of iron (II) and ammonium sulfate, (M); *V*_0_ is the volume of the sample to be analyzed; *V*_1_ is the volume of the solution of iron (II) and ammonium sulfate, used for the titration of the control sample, in milliliters; *V*_2_ is the volume of the iron (II) and ammonium sulfate solution, used for the titration of the sample to be analyzed, in milliliters; 8000 is the molar mass of 1/2 O_2_, in milligrams per liter.

### 3.3. Microscopic Examination of the Biological Sludge in the Visual Field Using Gram Staining and TEM Microscopy

A 1 mL aliquot volume was collected daily from the bioreactor after measuring the sludge volume. Microorganisms were identified by examining them under an optical microscope to estimate the population of germs in the microfauna of the pilot plant and to analyze the dynamics of the ciliate population in the tested biocenosis [51,52,53]. The microscopic examination was performed using a 40 × 12.5 objective lens, and staining smears were performed with a 90× immersion objective. The results obtained are presented in Table 1 and Table 2, and the estimation of the number of microorganisms is shown graphically. To visualize the biological composition of the harvested sludge in detail, samples were scanned using TEM.

### 3.4. Determination of the Total Number of Bacterial Colonies from the Samples Analyzed Through Culture Examination

During the experiment, daily, 1 mL of the biological sludge sample was taken from the bioreactor and diluted to a dilution of 10^−5^ in sterile ultrapure water [47,48,49].

Then, we took 1 mL of this dilution and inoculated it onto the agar medium. Each plate was then placed in a thermostat and incubated for 48 h at a temperature of 30–37 °C. After the 48 h incubation period, we counted the total number of resulting colonies according to Equation (8):

X (CFU/mL) = N × d × 1/v,(8)

where:

X = the number of colony-forming units,

N = the number of colonies counted on the plate,

d = the inverse of the dilution,

v = the inoculated volume.

## 4. Discussion

The physical processes that take place in the reactor are mass transfer of oxygen and substrate at the level of cells, of oxygen from air to water, adsorption of colloidal particles and fine suspensions on the biomass surface, desorption of metabolic products, gravitational sedimentation, etc. The existing chemical processes in the reactor can be maintained by hydrolysis reactions, oxidation-reduction reactions, dehydration reactions, precipitation and coagulation reactions, and pH modification. For the effectiveness of the purification process, the hydraulic processes must also be taken into account, including the flow regime, the distribution of the polyphase medium in the aeration basin, the hydraulic retention time, sedimentation speeds, hydraulic loads, convection and density currents, etc.

The biocenosis in the biological sludge consists of heterotrophic and autotrophic bacteria. The latter synthesize their energy from the oxidation of substances introduced into the system in the presence of oxygen introduced through the aeration process. The nitrification process is provided by autotrophic bacteria. The general oxidation reaction is presented in Equation (9). Activated sludge consists of microorganisms that develop in *Zooglee ramigera* that give complexity to the sludge flocs. The structure of the sludge flocs also depends on whether the bioreactor was fed continuously, with this leading to an increase in the mass of the biological material due to the diffusion of ions from the synthetic water introduced into the system [51,52,53,54,55,56].

2NH_3_ + 4O_2_ = 2NO_3_ + 2H^+^ + 2H_2_O (9)

Also, the diffusion of oxygen in the mass of the extracellular material helps to support the reactions involved in the purification process. The results obtained were a reflection of the experimental conditions, of the volume of active sludge obtained in the system during the experiments, of the diffusion of oxygen in the sludge floc, of the bacterial activity in the cellular material, of the C/N ratio in the system, and of the degradation reaction speed of the reactants inside the cells of the microorganisms.

For nitrification, the passage of nitrogen into ammoniacal nitrogen must precede the nitrification of NH_4_^+^, which is carried out in two stages: the oxidation of NH_4_^+^ into NO_2_^−^ and the oxidation of NO_2_^−^ into NO_3_^−^ thanks to nitrifying bacteria, which means that they ensure their growth by oxygenating mineral compounds. Denitrification takes place under the action of anaerobic heterotrophic microorganisms. The reaction that takes place is presented in Equation (10):

2NO_3_^−^ + 12H^+^ → N_2_ + 6H_2_O. (10)

In both stages of the experiment, we measured the pH and dissolved oxygen concentration daily using a WTW probe. The system was fed continuously in both phases. The optimal conditions regarding the pH for biological sludge is in the range of 6.5–8.5. The presence of inorganic substances in the feed water dosed with an increased flow would become harmful for the biological sludge. This is why a constant flow rate of 10 mL/h of synthetic water was adjusted, which did not change the pH of the biocenosis very consistently and ensured a natural ratio of biogenic elements in the biocenosis. In the first 10 days of the test period, the pH had lower values, in the range of 6.0–7.2, but the pH value of 6 did not significantly change the activity of microorganisms in the sludge flakes and did not negatively influence the presence and development of microorganisms. In the second half of the test period, after the 9th day, the pH was maintained in the value range of 7.4–8.0, which favored the appearance of the ciliates *Paramecium caudatum*, *Anabena*, *Nostoc*, and *Opercularia* as an indicator of stable water from the point of view of the chemical composition.

**Figure 2:**
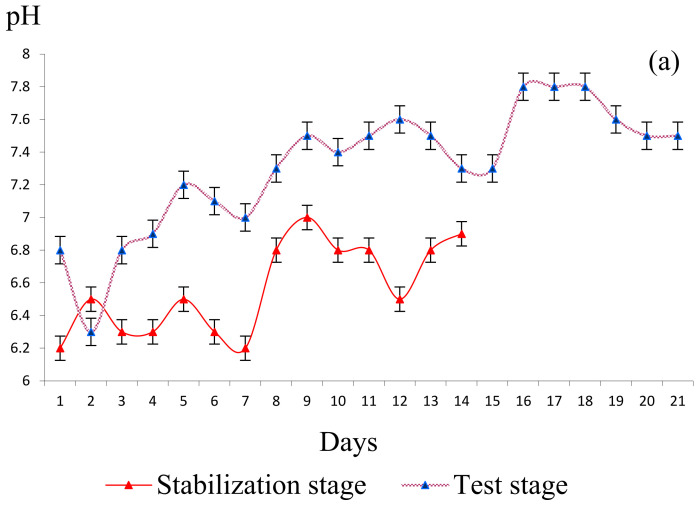

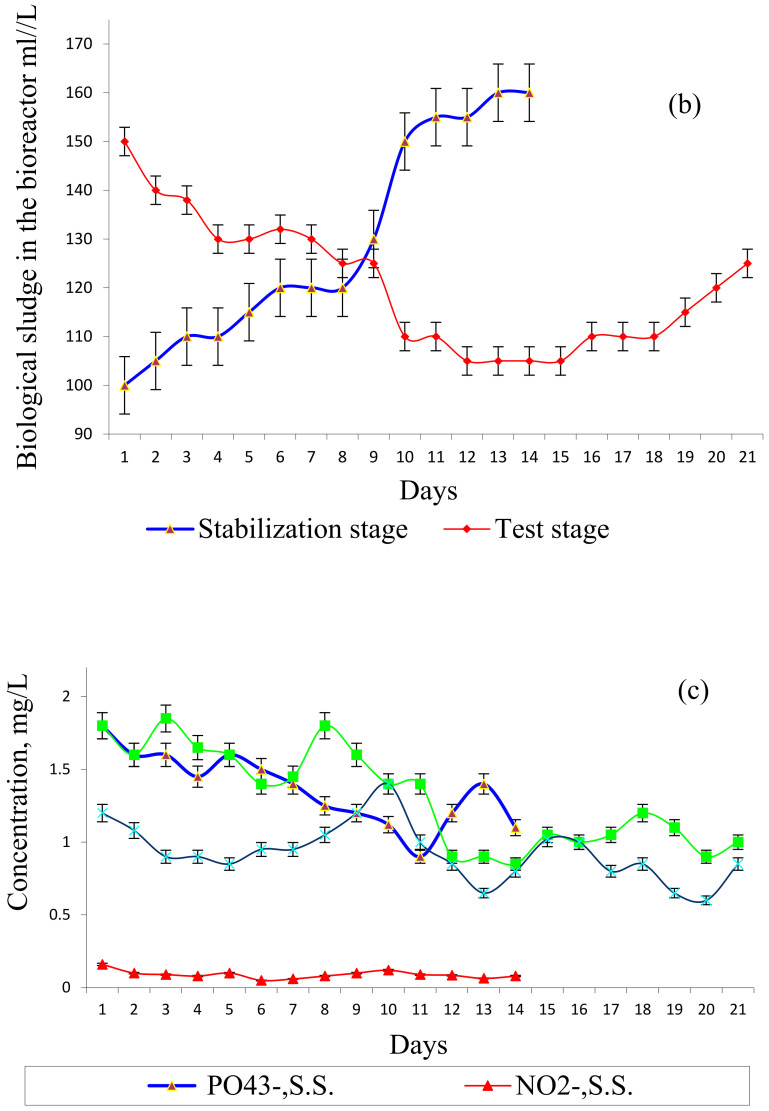

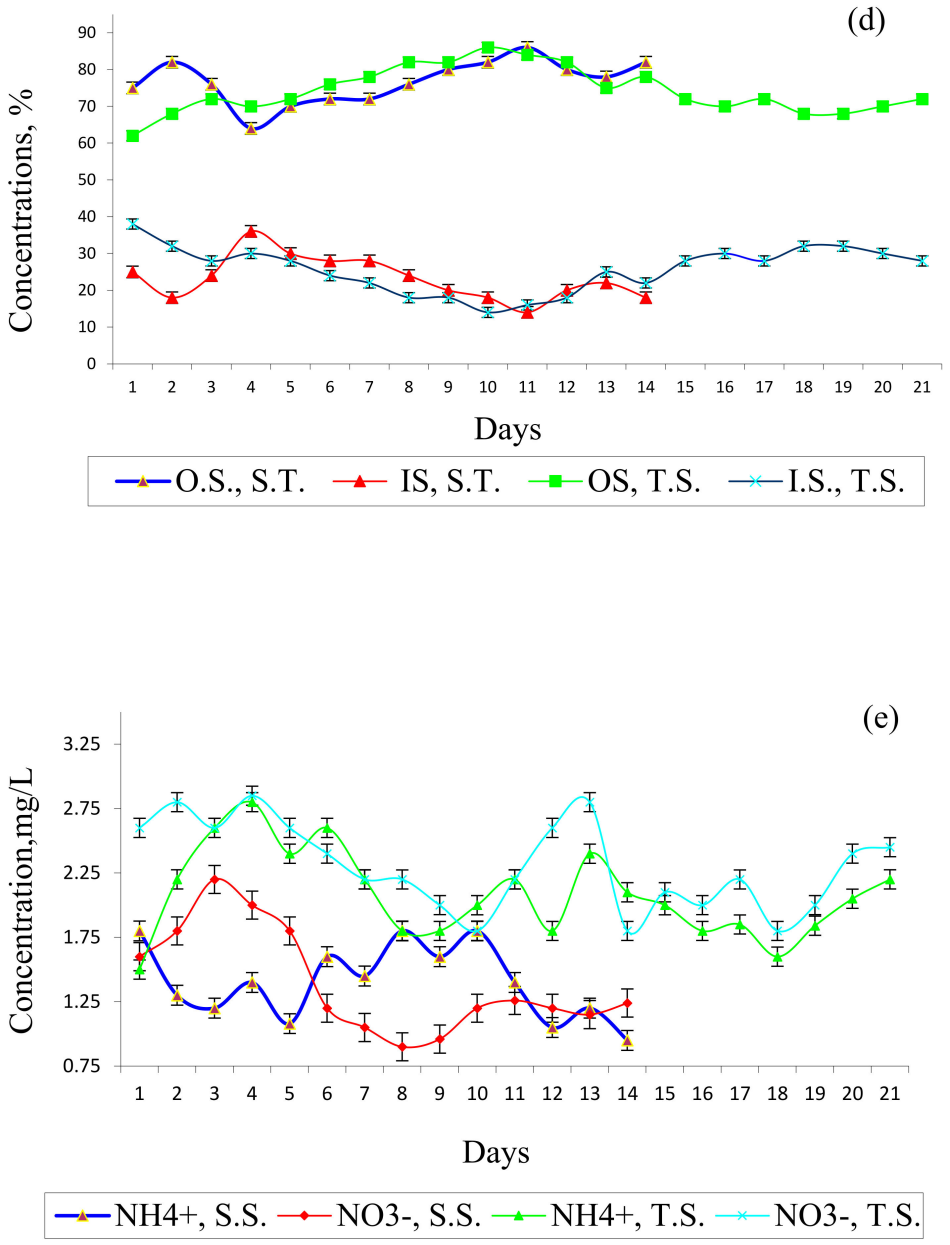
Experimental results obtained following the analysis of the sludge biological material in the stabilization stage (S.S.; 14 days) and in the test stage (T.S.; 21 days). (**a**) pH, (**b**) evolution of sludge volume in the bioreactor, (**c**) evolution of phosphate and nitrate concentrations, (**d**), organic and inorganic substances, and (**e**) ammonium and nitrate. Error bars represent standard deviation (*n* = 4).

**Figure 3:**
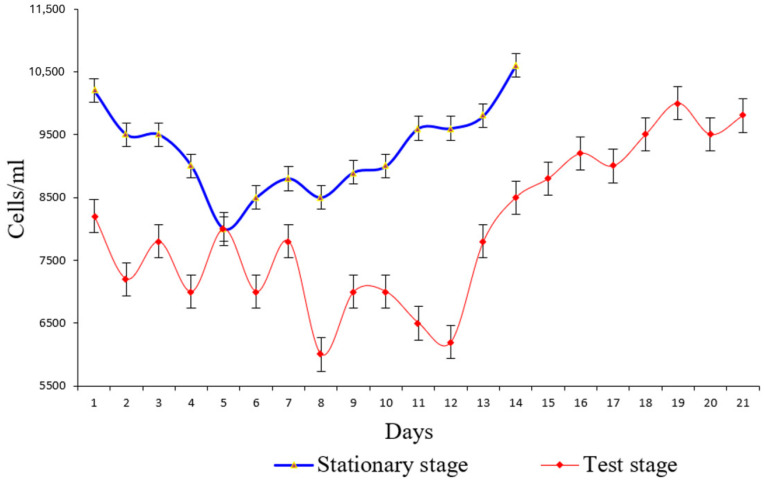
Experimental results regarding the number of microorganisms in the examined samples. Error bars represent standard deviation (*n* = 4).

Also, the inorganic ions introduced in the purification process were used as a source of carbon and energy for the synthesis of biological material. In this study, we followed the evolution of the microbiological composition in the biological sludge in the process of continuous treatment with synthetic water. The stability of the biological sludge was also monitored by measuring the volume of the sludge, which in the second half of the test stage was in mass expansion and grew and developed optimally, reaching a volume of 130 mL/L at the end of the experiment (Figure 2c). This was also highlighted by microscopic examinations regarding the number of cells/mL (Figure 3).

At certain time intervals each day, we stopped aeration for 30 min to allow the biological sludge in the aero tank to settle and measured the dynamics of the sludge volume. The experimental results are graphically represented in Figure 2a,b. Every day during both stages of the study, we conducted analyses to determine the chemical composition (NH_4_^+^, NO_3_^−^, NO_2_^−^, and PO_4_^3−^) by collecting the supernatant from the collecting vessel. The experimental results are shown in Figure 2c,e. The results regarding the biological and chemical composition of the biological sludge are shown in Figure 2d, and the number of microorganisms visualized is graphically quantified in Figure 3.

In the first two weeks of the biological sludge adaptation period in the laboratory, various types of protozoan and metazoan microorganisms were observed in the sludge sample [53]. The observations, detailed in Table 1 and Table 2, provide information about the biological state of the biological sludge (BS). The examination revealed the presence of free active ciliates *Aspidisca polystyla* and *Lyndonotus setigerum* (a predatory ciliate), which indicate a fresh biological sludge with low impurity. Additionally, the ciliate *Vorticella microstoma*, fixed by *Zooglee*, was identified, suggesting a favorable environment for life, low pollution, and good water circulation. The presence of the fixed ciliate *Opercularia* indicated an unpolluted water, this being a microorganism sensitive to pollution of the genus of green algae from the class *Chlorophyceae*. The mobile ciliates *Paramecium* sp. were also seen in abundance, which indicates an environment rich in nutrients, unpolluted, and adequately oxygenated. The ciliated microorganisms *Colpoda colpidium* and *Euplotes* also appeared in the examined samples. Predatory microorganisms (they feed on *Paramecium cells*) of the genus *Didinum nasutum* as well as trumpet-shaped ciliated microorganisms, *Stentor*, were also identified. Furthermore, from the class of suckers, the ciliate *Acineta tuberosa* was observed to be physed by *Zooglee*. Metazoa *Rotiferi* were visualized with reduced presence.

We stopped adapting the biological sludge at 14 days due to the fact that at that time, the viability of the microorganisms was very good, the bacterial cells were multiplying, and the eukaryotic microorganisms were present in considerable numbers. The presence of the microorganisms *Aspidisca polistila*, *Vorticella microstoma*, *Lytonotus setigerum*, *Acineta tuberosa*, *Paramecium caudatum*, *Opercularia*, *Nostoc*, *Anabena*, and *Rotiferi* was observed in the examined biological samples, and they are indicators of stable biocenoses, with the ability to biodegrade the chemical compounds introduced into the system. Regarding the denitrification capacity, it was ensured by the presence of the bacteria *Bacillus subtilis* and *Micrococcus* sp. These bacteria have the ability to reduce nitrates to nitrites and then nitrites to molecular nitrogen. Denitrification is ensured by the presence of the enzyme nitrate reductase contained in the bacterium *Bacillus subtilis* [57,58]. *Paramecium* are ciliates known to be a widely used organism in ecotoxicity tests due to their high resistance under toxic conditions, being an early warning bioindicator for environmental risks. These microorganisms have a cosmopolitan distribution and can be used to evaluate the toxicological effects of pollutants. *Bacillus subtilis* is very useful in the biodegradation processes of petroleum pollutants due to the fact that it has the ability to produce enzymes with which it can biodegrade and metabolize a wide variety of chemical compounds present in wastewater that can be difficult to remove and have a high degree of persistence (for example, petroleum hydrocarbons). It also has a role in denitrification processes [59,60].

BOD is a parameter that indicates cellular biochemical processes of aerobic microorganisms, such as oxidation of carbon and hydrogen (BOD-C) from organic substances used as a food source, oxidation of nitrogen (BOD-N) from nitrites, nitrates, and ammonium by microorganisms, as well as the oxidation of reducing substances, such as sulfite, ferrous, sulfide ions, etc., which chemically react with molecular oxygen. Due to the fact that in the analysis of biological oxygen demand (BOD), the biochemical and biological processes in the cells of microorganisms are involved, it must also be taken into account that the nitrification process is also involved in this analysis, as well as the heterogeneity of the population of microorganisms (the ratio between bacteria and protozoa), the carbon source from the reaction medium, etc.

The transition from the metabolism of organic substances that are soluble in the analyzed water to the metabolism of organic substances synthesized in the cellular mass can generate a plateau in terms of oxygen consumption. For example, protozoa use carbon to synthesize cell mass, a process called BOD-C. The biochemical reactions inside the biomass are reactions of biochemical oxidation of the substrate, growth of biomass, endogenous respiration, and inhibition of enzymatic reactions. Water loaded with organic pollutants can be sequentially attacked by microorganisms, which generates different biodegradation mechanisms and speeds depending on the dynamics of the biocenosis. COD is the amount of oxygen (measured in mg) consumed in one liter of water by oxidizable materials, under the action of an energetic chemical oxidant. The biological process is influenced by the following factors: temperature, pH, oxygen, time of passage, or contact of the bioreactor, which carries out the biological process in accordance with the hydrodynamic conditions of the process—mixing and homogenization. The ratio between BOD and COD assesses the quality of water and evaluates its toxicity. A ratio between 0.5 and 1 indicates a good treatability of the water, a ratio between 0.2 and 0.5 indicates the need to adapt the flora to the used water, and a ratio lower than 0.2 indicates the refractoriness of the used water to biological purification or its toxicity.

**Figure 4:**
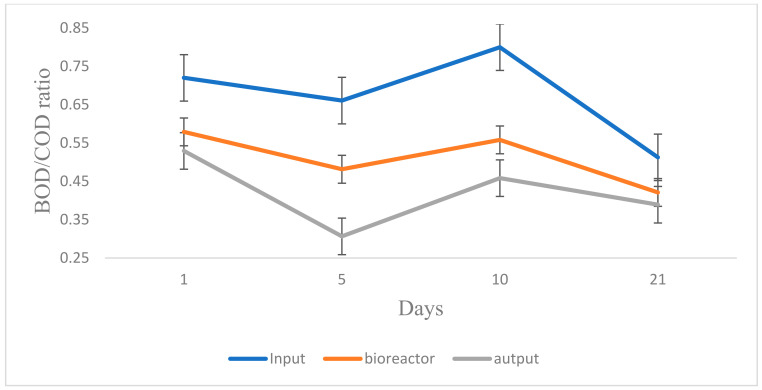
Variations in the BOD/COD ratio in the water treatment plant. Error bars represent standard deviation (*n* = 4).

The oxygen content in the water is proportional to the amount of biologically oxidizable organic matter contained in the water and, therefore, to its level of pollution. In the micro-pilot installation, we analyzed this report in different stages of the test (second test phase) and followed the content of COD and BOD in the samples collected in the feed vessel, in the bioreactor, in the decanter, and in the collector vessel. The BOD/COD ratios obtained at different times, taking into account the hydraulic retention time of the water used in the installation, are shown in Figure 4. A water whose BOD/COD ratio is higher than 0.3 is considered to be biodegradable. In the case of the experiment, we can say that the ratio was between 0.3 and 0.5 in the collector vessel of the installation, which means that the water is considered to be purified. In the composition of the mixed population of microorganisms, the development of the biocenosis depends on the environmental factors, on the metabolites released from the cellular processes, and on the simultaneous existence of a number of factors, such as the chemical composition of the water introduced into the system, the pH, the supply flow, and the chemical nature of the substrate. The development of the biocenosis of the Zoogleal substrate protects the bacterial species from the attack of predatory ciliates and favors the absorption of nutrients [61,62,63,64].

**Figure 5:**
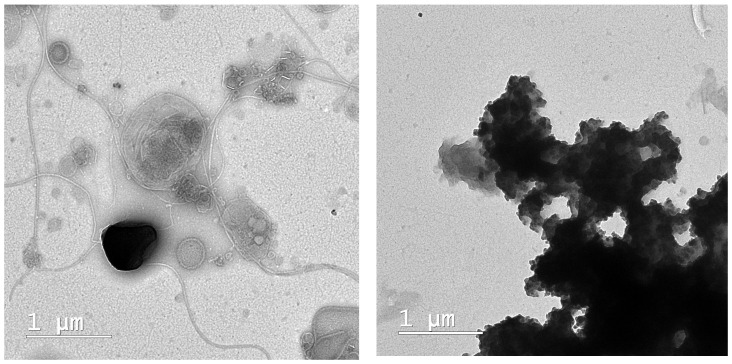

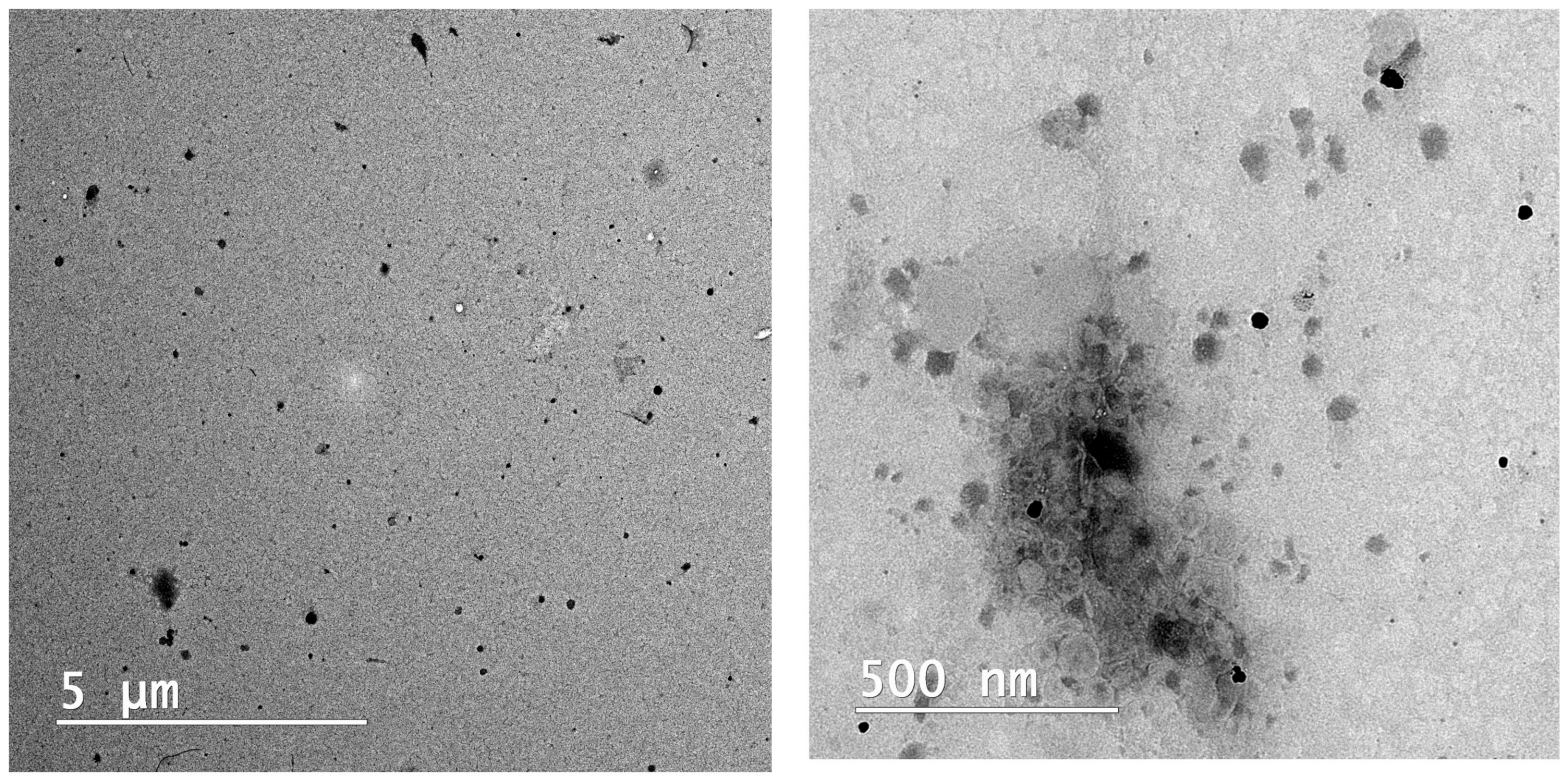
TEM images of the biological sludge collected from the bioreactor in the test phase.

The biological suspension collected from the bioreactor was examined using TEM. The results obtained are presented in Figure 5. The Gram-stained smear analysis revealed the presence of abundant, well-bound *Zooglee ramigera*, cocci, and bacilli, both Gram-positive and Gram-negative bacteria. Filamentous algae, *Nostoc* and *Anabena* bacteria, and colonial and solitary microorganisms were also detected. These microorganisms serve as environmental indicators of sufficient nutrient levels. They consist of various types of protists and cyanobacteria, which can be visualized through microscopic examination.

**Figure 6:**
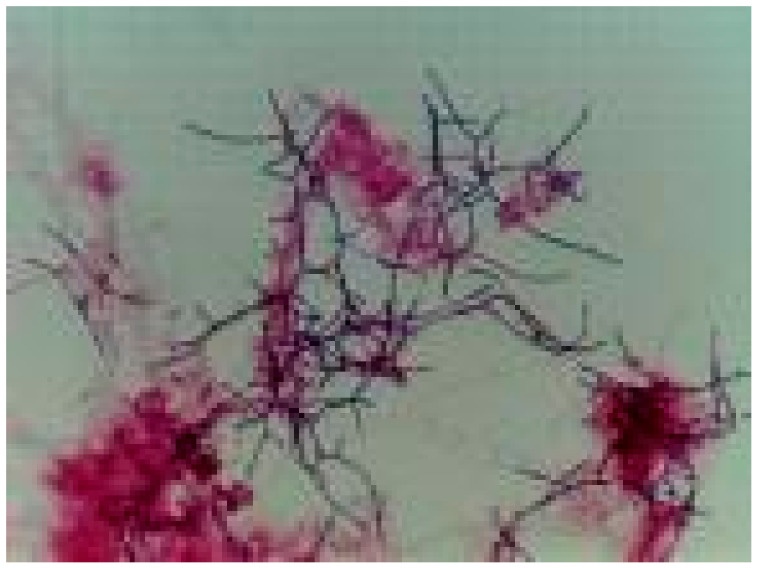
Microscopic aspect—*Nocardia* image obtained by Gram-staining smear in the test phase.

Protozoa are single-celled microorganisms that are crucial in aquatic ecosystems. They play a key role in breaking down organic compounds through various mechanisms. In biological sludge, the food chain of microorganisms operates on the basis of protozoa’s ability to consume bacteria, algae, and other microscopic microorganisms. By controlling bacterial populations, protozoa have the ability to impact bacterial activity in the bioreactor environment and can stimulate the growth and activity of bacteria involved in the decomposition of organic compounds. When protozoa consume microorganisms, they indirectly contribute to the breakdown of pollutants in the aquatic environment by converting organic toxins into simpler metabolic products. This creates substances that can be further degraded by other microorganisms. Additionally, protozoa aid in nutrient recycling by consuming bacteria and algae, thus facilitating the transformation of complex organic substances into forms that are more easily utilized by other microorganisms in nutrient cycles. They also play a role in balancing the diversity of biosystem communities and preventing the excessive proliferation of bacterial species that could hinder the decomposition of organic material. To identify specific microbial strains in biological sludge suspension, it was necessary to prepare individual culture media for each bacterium analyzed [35,36,37,38,39,40,41,42]. The total number of bacterial colonies was determined by counting the colony-forming units (CFU) that developed on the agar culture medium after samples were harvested and dilutions were performed. According to the calculations based on Equation (5), the experimental results indicated values between 8 × 10^8^ and 6 × 10^10^ CFU/mL in the biological sludge stabilization stage, and between 2 × 10^9^ and 1.2 × 10^10^ in the testing stage. Bacterial cultures were developed on agar medium, and passages were conducted on specific solid culture mediums for each bacterium. Cultural characters were assessed after 24–48 h of incubation at 30–37 °C in a thermostat. Identification tests were performed for *Microccocus luteus*, *Bacillus subtilis*, and *Nocardia* bacteria. Gram-staining smears were made from each pure culture obtained after selecting the culture media, and these were visualized under the optical microscope with a U I90 x 1.25 immersion objective. Furthermore, the cultures on the agar medium were used to count the total number of bacteria in the examined biological sludge, followed by transitions to medium C. Incubation at 30–37 °C for 24–48 h was then conducted. Additionally, a culture medium supplemented with lysostaphin (200 µg/mL) was prepared to inhibit the growth of staphylococci and encourage the development of micrococci. Following this, passages were performed using a loop (it is passed with the loop in a zigzag) on medium A and medium B, and incubation occurred at 30–37 °C for 24 h. The appearance of yellow colonies on medium A and golden-yellow colonies on medium B suggested the presence of *Micrococcus luteus* bacteria. Identification elements for *Micrococcus luteus* included the appearance of round, convex colonies with regular edges and various pigmentation. Gram-positive smears revealed spherical cocci arranged in tetrads or irregular piles. Transitions from the cultures on the nutrient agar medium to the specific medium for *Bacillus subtilis* were performed. Incubation at 30 °C for 24–48 h resulted in the appearance of large colonies with a matte, opaque surface, colored in cream and brown. Morphological examination revealed small, Gram-positive bacilli, isolated or grouped in short chains, indicating the presence of *Bacillus subtilis*. Finally, transitions from the cultures on the nutrient agar medium to the specific medium for *Nocardia* bacteria were performed. Following incubation at 30–37 °C for 24–48 h, small colonies appeared on the culture medium, which were then incubated for an additional 3–7 days [48,49,50,64,65,66]. The cultural and microscopic examinations revealed areas adherent to the environment due to the development of submerged, smooth, irregular hyphae, indicating the presence of *Nocardia* bacteria in the form of thin filaments (Figure 6).

## 5. Conclusions

In biological wastewater treatment, understanding the tolerance limits of the microorganisms is necessary. This study focused on the chemical and biological composition of the biological sludge from the petrochemical industry under laboratory conditions in the micro-pilot plant to evaluate the optimal conditions for the viability of microorganisms.

The appearance of protozoa and metazoa in the analyzed biocenosis indicated an active biomass, with these microorganisms being considered an indicator of purification in optimal living conditions. Due to the oxygenation conditions of the biocenosis, maintaining an optimal pH between 6.5 and 8.5, with the presence of nutrients and undesirable substances in the nutrient substrate, it can be concluded that during the experiment, after the seven-day test period, the biocenosis indicated a stable, balanced biomass from the point of view of the microbiological composition of the activated sludge. Selective media highlighted the presence of bacteria, such as *Bacillus subtilis*, *Micrococcus luteus*, and *Nocardia*, while microscopic examinations revealed the presence of ciliates, including *Paramecium caudatum*, *Stentor*, *Vorticella microstoma*, *Aspidisca polistila*, *Opercularia*, *Acinetta tuberosa*, *Lychnothamnus setigerus*, *Nostoc*, and *Anabena*, as well as metazoans of the *Rotifer* type. The biocenosis was found to adapt to the introduction of pollutants through synthetic water into the biological sludge purification system, biodegrading the pollutants and using them as a food source. The diversity of microorganisms present in the activated sludge requires the continuation of studies regarding the degree of survival of the microbiological population from the biocenosis in the presence of potentially toxic substances to study the inhibition generated by them. It is also necessary to study other species of microorganisms, bacteria, ciliates, protozoa, and metazoa in the presence of concentrations of pollutants.

## Figures and Tables

**Table 1:** Experimental results regarding the observation of microorganism species in the biological sludge, analyzed during the stabilization period of the biological sludge.

Microorganisms Visualized by Microscopic Examination	Adaptation of Sludge to Laboratory Conditions					
	Sample A	Sample B	Sample C	Sample D	Sample E	Sample F
*Aspidisca polistila*	+	+	+	+	+	+
*Vorticella microstoma*	+	+	+	+	+	+
*Lytonotus setigerum*	+	-	-	-	+	+
*Acineta tuberosa*	-	-	-	+	+	+
*Paramecium caudatum*	+	+	+	+	+	+
*Didinum nasutum*	-	+	+	+	-	-
*Stentor*	-	+	+	+	-	-
*Opercularia*	-	-	+	+	+	+
*Nostoc*	+	-	-	+	+	+
*Anabena*	+	-	-	+	+	+
*Colpoda colpidium*	+	+	+	-	-	-
*Euplotes*	-	+	+	+	-	-
*Rotiferi*	+	+	+	+	+	+

+, Observation of the microorganism in >50% of all samples examined; -, lack of the microorganism in the microscopically examined samples. The samples were collected in the stabilization phase: A = first day of collection, B = after 3 days, C = after 6 days, E = after 10 days, and F = after 14 days.

**Table 2:** Experimental results of the microorganism species in the biological sludge analyzed in the testing stage.

Microorganisms Visualized by Microscopic Examination	Date of Analysis of Samples			
	Sample A	Sample B	Sample C	Sample D
*Aspidisca polistila*	+	+	+	+
*Vorticella microstoma*	+	+	+	+
*Lytonotus setigerum*	+	-	-	-
*Acineta tuberosa*	-	-	-	+
*Paramecium caudatum*	+	+	+	+
*Didinum nasutum*	-	+	+	+
*Stentor*	-	+	+	+
*Opercularia*	-	-	+	+
*Nostoc*	+	-	-	+
*Anabena*	+	-	-	+
*Colpoda colpidium*	+	+	+	-
*Euplotes*	-	+	+	+
*Rotiferi*	+	+	+	+

+, Observation of the microorganism in >50% of all samples examined; -, lack of the microorganism in the microscopically examined samples. The samples were collected in the testing phase: A = first day of treatment with synthetic water in the testing phase, B = after 7 days of testing, C = after 14 days of testing, and D = after 21 days of testing.

## Data Availability

The data presented in this study are available on request from the corresponding author.
